# A Treatise on the Science and Practice of Midwifery

**Published:** 1889-11

**Authors:** 


**A Treatise on the Science and Practice of Midwifery.** By W. S. PLAYFAIR, M. D., LL.D., F. R. C. P., Professor of Obstetric Medicine in King's College; Physician for the Diseases of Women and Children to King's College Hospital, etc., etc. Fifth American, from the seventh English, edition, with notes and additions by ROBERT P. HARRIS, M. D., with five plates and 207 illustrations. Philadelphia: Lea Brothers & Co. 1889.

A new edition of this standard work on the science and practice of obstetrics "sufficiently Americanized upon the points where English and American obstetricians differ in opinion and practice, to fit it for the uses of American students and obstetricians," will be hailed with pleasure by all who are interested in this department of medicine. Playfair's work has always been favorably received by the profession, and its conservatism has made its teachings a safe guide in obstetric procedures. The author has the merit of conciseness and clearness, which makes this work a *multum in parvo*, and, therefore, of special value to the busy physician.

The editor in this edition has added the obstetric nomenclature adopted at the International Medical Congress, held in Washington in 1887, which we regard with special favor, inasmuch as it will lead to uniformity in obstetric terms—a result to be desired by all students.

The Porro-Cesarean operation, the conservative Cesarean operation, and the recent advances made in the treatment of extra-uterine pregnancy, are subjects which the editor in this edition has brought down to the close of the year 1888, while many of the American additions have been either rewritten or remodeled, and many new short notes added when required to clear up the various subjects treated.

New illustrations and plates have been added, making the work more valuable to students especially, than the previous editions.

Upon the whole, this edition has been greatly improved over those preceding, and the work may well rank among the best offered to the student or practitioner. The American editor has performed the task assigned him with good judgment and ability, and Playfair's Obstetrics, under the auspices of the great publishing house that issues it, will maintain the high reputation which its merits and conservatism well deserve.

